# Estimation of heritabilities and genetic correlations by time slices using predictivity in large genomic models

**DOI:** 10.1093/genetics/iyaf066

**Published:** 2025-04-05

**Authors:** Ignacy Misztal, Gopal Gowane

**Affiliations:** Animal and Dairy Science, University of Georgia, Athens, GA 30602, USA; On leave from Animal Genetics and Breding Division, ICAR-National Dairy Research Institute, Karnal 132001, India

**Keywords:** genetic parameters, genomic prediction, correlated response, genomic selection, parameter estimation, large data, heritability, genetic correlation, predictability

## Abstract

Under genomic selection, genetic parameters may change rapidly from generation to generation. Unless genetic parameters used for a selection index are current, the expected genetic gain may be unrealistic, possibly with a decline for antagonistic traits. Existing methods for parameter estimation are computationally unfeasible with large genomic data. We present formulas for estimating heritabilities and genetic correlations applicable for large models with any number of genotyped individuals. Heritabilities are calculated by combining 2 formulas for genomic accuracies: one that relies on predictivity and another that depends on the number of independent chromosome segments. Genetic correlations are calculated from predictivities across traits. We simulated data including 2 traits for 240,000 genotyped and phenotyped animals in 6 generations, namely, production trait with an initial heritability of 0.4 and a fitness trait with a fixed heritability set at 0.1 in each generation. Only the first trait (production) was selected, whereas the second trait (fitness) was constructed so that its genetic correlation with the first trait declined by about 0.1 per generation. Calculations were for 3-generation windows, with the first 2 generations treated as a reference population. Compared with realized values, the estimated heritabilities were within 0.02. Genetic correlations were within 0.15 with predictivity of production phenotype by prediction for fitness and within 0.05 with predictivity of the fitness phenotype by prediction for production. The proposed formulas enable the estimation of heritabilities and genetic correlations by time slices for models in which predictivities can be calculated and genetic evaluation is feasible.

## Introduction

Genomic selection is now widely practiced across the breeding industry, as evidenced by large-scale genotyping using inexpensive SNP chips. For example, as of March of 2024, genotypes were available for over 7 million US Holsteins (https://queries.uscdcb.com/Genotype/cur_freq.html), over 1 million American Angus (www.angus.org), and over 200,000 animals per line for some pig and broiler breeding companies. Because the desired outcome of selection in animal populations is a balanced animal, selection indices involve many traits (e.g. VanRaden *et al*. 2021) and are derived from estimated genetic parameters.

Various studies conducted before selection could be based on genomic information indicated only slow changes in genetic parameters over time (Tsuruta *et al*. 2004; Sosa-Madrid *et al*. 2023). After genomic selection became feasible, however, rapid changes in these parameters were reported by Hidalgo *et al*. (2020) in a commercial pig population. Over a few years, the heritability of a growth trait halved, whereas its correlation with a fitness trait grew 50% more antagonistic. Similar changes under genomic selection have recently been detected in broiler chickens by Sosa-Madrid *et al*. (2023) and Richter *et al*. (2024). The estimation by Sosa-Madrid *et al*. (2023) ignored genomic information, whereas the other studies presented estimates both with and without genomic information. When genomic information was considered, the changes were stronger for heritabilities but weaker for genetic correlations (Hidalgo *et al*. 2020). All of these calculations, especially those using genomic information, were computationally intensive.

Accurate genetic parameters are essential for realistic trait trends and setting up a selection index (Hazel 1943; Hazel *et al*. 1994). With rapidly changing parameters, trends derived using old parameters may be exaggerated and lead to unrealistic estimates of genetic gain. A selection index constructed with old parameters may lead to the decline of important fitness traits. If such declines are not addressed quickly by a change in the selection index, a reversal may take a long time because of the low heritabilities of fitness traits.

Genetic parameters may change with selection for a variety of reasons, such as the Bulmer effect and drift (Bulmer 1971; Macedo *et al*. 2020), different definitions of traits over time (Tsuruta *et al*. 2004), nonadditive effects (Duenk *et al*. 2020), metabolic stress from changing resource allocation (van der Waaij 2004), and nonlinearity among traits (Fuerst-Waltl *et al*. 1998). The Bulmer effect is perhaps the primary mechanism behind the rapid change in heritabilities for traits with higher heritability because the genetic pressure for those traits accelerates under genomic selection. The rapid changes in genetic correlations between production and fitness traits may result from a redefinition of these traits due to changes in nutritional resource allocation (Tsuruta *et al*. 2004; van der Waaij 2004), whereas changes under a regular additive model would be slow.

One example of a trait redefinition is productive life, causing its genetic correlation with dairy form (thinness) in Holstein dairy cattle to change from positive to negative (Tsuruta *et al*. 2004). Earlier, when cows produced less, poor dairy form indicated that cows spent energy on getting fat instead of producing milk. When cows began to produce more, body fat was required to compensate for the negative energy balance in mid-lactation. Although productive life was initially positively correlated with dairy form, the correlation was later changed to negative. Genetic models that consider nutritional resource allocation are complex and not yet suitable for routine use (Bouquet *et al*. 2022).

Common methods for parameter estimation, such as restricted maximum likelihood (REML) or Bayesian via Gibbs sampling, are used to estimate genetic parameters for a base population. Several studies have proposed modified methods or models for estimation by time intervals. Sorenson *et al*. (2001) presented a method to calculate variances by generation using Bayesian models with 90 samplings separately for each generation. Macedo et al. (2020) used Sorensen's method to analyze data from 540,000 sheep over 40 years without including any genomic information. Lara *et al*. (2022) applied a Bayesian method to marker models with simulated data for about 3,000 subjects. Gianola *et al*. (2022) revisited various issues arising from selection with genomic data. Tsuruta *et al*. (2004) looked at changes in genetic parameters in Holsteins using 3 approaches: a multiple-trait model in which each trait contained phenotypes from a time slice, a random-regression model with regression on birth year, and models based on separate time slices. All models had to use data samples because the complete data set was computationally too large. Estimates from the random-regression model were smoother because of the constraints of the linear regression, and estimates from single-trait models with times slices were more variable. Haile-Mariam and Pryce (2015) used methods from the Tsuruta study for Australian Holsteins. Van der Waaij (2004) simulated data with special accounting for metabolic stress. Although the parameters showed large changes over the selection interval, parameters were not estimated but derived directly from simulated data.

Estimating parameters with genomic information by established methods is computationally expensive because genomic matrices are dense (Misztal *et al*. 2021). With REML, computations increase cubically with the number of genotyped individuals (Masuda *et al*. 2015). Although parameters are usually estimated using data samples, this could lead to biases under genomic selection because of unaccounted selection (Cesarani *et al*. 2021). Gao *et al*. (2019) applied REML to simulated populations and observed biases with selective genotyping. In selective genotyping, in which young animals are mass-selected for growth and only the best are genotyped, the estimate of heritability without genomic information is about 0.3 but reaches 0.8 with genomic information (Wang *et al*. 2020). In a broiler population, the same inflation for a single trait was observed (Richter *et al*. 2024).

Analyses by Hidalgo *et al*. (2021), who used a Bayesian estimation with Gibbs sampling, required compromises. Because a random-regression model was too expensive computationally, estimation was by time slices. Computations took several months for a limited number of traits. Although their model included 39,000 genotyped animals, the breeding organizations routinely have over 200,000 genotyped animals per line. One possibility for more efficient computing would be marker models (Fernando *et al*. 2014; Lee and van der Werf 2016; Lara *et al*. 2022) because the number of markers is irrespective of the number of animals. However, the limitation in REML does not arise from genomic relationships but from pedigree relationships for genotyped individuals (Junqueira *et al*. 2022). In addition, published studies on estimating parameters with SNP models and large data sets are scarce.

Developing alternative methods for estimating current parameters is desirable because the adverse effects of using old parameters may be large. Such alternatives would provide estimates for the last generation with all applicable data, including genomic, and would be inexpensive to run routinely. The simplest method applicable to large data sets is treating genomic estimated breeding values (GEBVs) as true breeding values and estimating parameters directly from GEBVs. Such a method may be satisfactory if the accuracies of GEBVs are close to 1. Another possibility is to exploit 2 formulas for genomic accuracy.

Legarra *et al*. (2008) showed that predictivity [correlations of estimated breeding values (EBVs) with future phenotypes adjusted for fixed effects] is a function of heritability and accuracy of EBVs. If heritability is known, accuracy can be estimated. Conversely, if accuracy is known, heritability can be estimated. Misztal *et al*. (2023) compared estimates of accuracy obtained from predictivity and from a formula based on independent chromosome segments and population details (Daetwyler *et al*. 2008). Both formulas agreed (within 0.02) except for a case in which initial heritability was inflated.

The predictivity formula requires adjusted phenotypes for the animal as well as GEBV for 1 trait. If predictivity is across traits, the formula may also include their genetic correlation. The purpose of this paper is 3-fold: firstly, to develop a new method to estimate heritabilities in time slices based on predictivity; secondly, to determine if across-trait predictivity can be used to estimate genetic correlations in time slices; and finally, to test the developed formulas with large data sets.

## Methods

### Predictivity and theoretical accuracies

Assume a model


(1) y=Xb+u+e,

where **y** is a vector of observations, **X** is a design matrix, **b** is a vector of fixed effects, **u** is a vector of random effects, and **e** is a vector of residuals. Legarra *et al*. (2008) showed that the average accuracy of an evaluation (*acc*) for selected animals could be calculated from predictivity, a correlation between their adjusted phenotypes and EBVs:


(2) $$acc=\frac{\mathrm{Corr}(y-Xb,\;\hat{u})}{h},$$

where **y** − **Xb** is a vector of observations for *n* selection candidates (the validation population) adjusted for fixed effects, $\hat{u}$ is a vector of EBVs for those animals calculated from *N* observations excluding the validation population, and *h*^2^ is heritability. Daetwyler *et al*. (2008) proposed a formula for *acc* in a population with *M_e_* independent chromosome segments, *N* genotyped and phenotyped animals, and heritability *h*^2^:


(3) $$acc=\sqrt{\frac{Nh^{2}}{Nh^{2}+M_{e}}}.$$

If estimates of *M_e_* are available (e.g. from Pocrnic *et al*. 2016a), an estimate of *h*^2^ ($\hat{h^{2}}$) can be derived by equating both formulas:


(4) $$\hat{h^{2}}:\sqrt{\frac{Nh^{2}}{Nh^{2}+M_{e}}}=\frac{\mathrm{Corr}(y-Xb,\;\hat{u})}{h}.$$

Then,


(5) $$\hat{h^{2}}=\frac{c^{2}+\sqrt{c^{4}+\frac{4c^{2}M_{e}}{N}}}{2},$$

where $c=\mathrm{Corr}(y-Xb,\;\hat{u})$. In practice, the true value of fixed effects **b** is unknown and is replaced by an estimate.

### Predictivity across traits and genetic correlations

Predictivity can be generalized to estimate genetic correlations. Assume a 2-trait model with *i* and *j* indicating two traits:


(6) $$\begin{array}{rl} & y_{i}=X_{i}b_{i}+u_{i}+e_{i};\\ & y_{j}=X_{j}b_{j}+u_{j}+e_{j}, \end{array}$$

with variances


(7) $$\begin{array}{rl} & \mathrm{Var}\left(\left[\begin{array}{c} u_{i}\\ u_{j} \end{array}\right]\right)=G⊗\left[\begin{array}{cc} \sigma_{u_{i}}^{2} & \sigma_{u_{ij}}\\ \sigma_{u_{ij}} & \sigma_{u_{j}}^{2} \end{array}\right];\\ & \mathrm{Var}\left(\left[\begin{array}{c} e_{i}\\ e_{j} \end{array}\right]\right)=I⊗\left[\begin{array}{cc} \sigma_{e_{i}}^{2} & 0\\ 0 & \sigma_{e_{j}}^{2} \end{array}\right], \end{array}$$

where **G** is a relationship matrix; $\sigma_{u_{i}}^{2}$ and $\sigma_{u_{j}}^{2}$ are additive variances for traits *i* and *j*, respectively; $\sigma_{u_{ij}}$ is covariance between the traits; and $\sigma_{e_{i}}^{2}$ and $\sigma_{e_{j}}^{2}$ are residual variances. Based on the conditional distribution $u_{i}|u_{j}$,


(8) $$u_{i}|u_{j}=\frac{\sigma_{u_{ij}}}{\sigma_{u_{j}}^{2}}u_{j}+\varepsilon ,$$

where **ε** is a vector of uncorrelated random values, the predictivity of the adjusted phenotype of trait *i* for EBV of trait *j* is


(9) $$\begin{array}{rl} & \begin{array}{c} \mathrm{Corr}(y_{i}-Xb_{i},{\hat{u}}_{j})=\frac{\mathrm{Cov}(u_{i},{\hat{u}}_{j}\;)}{\sqrt{\sigma_{\;p_{i}}^{2}\sigma_{{\hat{u}}_{j}}^{2}}}=\frac{\frac{\sigma_{u_{ij}}}{\sigma_{u_{j}}^{2}}\mathrm{Cov}(u_{j},{\hat{u}}_{j})}{\sqrt{\sigma_{\;p_{i}}^{2}\sigma_{{\hat{u}}_{j}}^{2}}} \end{array}\\ & \;=\frac{\frac{\sigma_{u_{ij}}}{\sigma_{u_{j}}^{2}}\mathrm{Cov}(u_{j},{\hat{u}}_{j})}{\sqrt{\frac{\sigma_{u_{i}}^{2}}{\sigma_{u_{i}}^{2}}\frac{\sigma_{u_{j}}^{2}}{\sigma_{u_{j}}^{2}}\frac{\sigma_{u_{j}}^{2}}{\sigma_{u_{j}}^{2}}\sigma_{\;p_{i}}^{2}\sigma_{{\hat{u}}_{j}}^{2}}}=\frac{\frac{\sigma_{u_{ij}}}{\sigma_{u_{j}}^{2}}\mathrm{Cov}(u_{j},{\hat{u}}_{j})}{\frac{\sqrt{\sigma_{u_{i}}^{2}\sigma_{u_{j}}^{2}}}{\sigma_{u_{j}}^{2}}\sqrt{\frac{\sigma_{\;p_{i}}^{2}}{\sigma_{u_{i}}^{2}}\;\;}\sqrt{\sigma_{u_{j}}^{2}\sigma_{{\hat{u}}_{j}}^{2}}}\\ & \;=\frac{\frac{\sigma_{u_{ij}}}{\sigma_{u_{j}}^{2}}}{\frac{\sqrt{\sigma_{u_{i}}^{2}\sigma_{u_{j}}^{2}}}{\sigma_{u_{j}}^{2}}}\frac{\mathrm{Cov}(u_{j},{\hat{u}}_{j})\;}{\sqrt{\sigma_{u_{j}}^{2}\sigma_{{\hat{u}}_{j}}^{2}}}\sqrt{\frac{\sigma_{u_{i}}^{2}}{\sigma_{\;p_{i}}^{2}}\;\;}=Corr(u_{i},u_{j})\;acc_{j}\;h_{i} \end{array}$$

where $acc_{i},\;\sigma_{\;p_{i}}^{2}$, and $h_{i}$ are accuracy, phenotypic variance, and square root of heritability of trait *i*, respectively. After rearranging, the genetic correlation between traits *i* and *j* is


(10) $$\begin{array}{c} Corr(u_{i},u_{j})\;=\frac{\mathrm{Corr}(y_{i}-Xb_{i},\;{\hat{u}}_{j})}{h_{i}acc_{j}}. \end{array}$$

Under regular models such as equation (7), Corr(u_i,u_j)=Corr(u_j,u_i), and asymptotically


(11) $$\begin{array}{c} \frac{\mathrm{Corr}(y_{i}-Xb_{i},\;{\hat{u}}_{j})}{h_{i}acc_{j}}=\;\frac{\mathrm{Corr}(y_{j}-Xb_{j},\;{\hat{u}}_{i})}{h_{j}acc_{i}}, \end{array}$$

although the standard errors may differ.

### Standard errors

For two n×1 random variables from the normal distribution, the standard error (SE) of correlation *ρ* is $(1-\rho^{2})/\sqrt{n-3}$ (Gnambs 2023). For large *n*, SE of *ρ* is <1/ $\sqrt{n}$. Then, treating $h_{i}$ as constant, SE in the accuracy formula is


(12) $$\begin{array}{c} SE(acc)<\frac{1}{h_{i}\;\sqrt{n}\;}. \end{array}$$

Assume a trait with a heritability of 0.25 and 1,600 observations. Then SE(acc) would be <0.05 with a 95% confidence interval of <0.2 wide. The error would decrease with a larger validation population and a higher heritability.

The SE in the genetic correlation formula can be calculated by treating *h_i_* and *acc_j_* as constants. Then


(13) $$\begin{array}{c} SE[\mathrm{Corr}(u_{i},u_{j})]\;<\frac{1}{h_{i}acc_{j}\sqrt{n}\;}.\; \end{array}$$

Assume 2 traits with heritabilities of 0.25, EBVs with accuracies of 0.5, and 1,600 animals in the validation population. Then SE would be <0.10 with a 95% confidence interval of <0.4 wide. The error would decrease with a larger validation population, higher heritability of the first trait, and higher accuracy of the second trait. Note that the SE generally would be different for $\mathrm{Corr}(u_{i},u_{j})\;\mathrm{and}\;\mathrm{Corr}(u_{j},u_{i})\;$ and it would be smaller for a combination with the higher denominator.

An approximate SE for $\hat{h^{2}}$ can be calculated based on a linear approximation:


(14) $$\begin{array}{r} \begin{array}{c} SE({\hat{h}}^{2})\approx \frac{d{\hat{h}}^{2}\;}{dc}SD(c)\approx \frac{1}{2\sqrt{n}\;}\left[c+\frac{2c^{3}+\frac{4cM_{e}}{N}}{\sqrt{c^{4}+\frac{4c^{2}M_{e}}{N}}}\right]\\ =\frac{1}{2\sqrt{n}\;}\left[c+\frac{2c^{2}+\frac{4M_{e}}{N}}{\sqrt{c^{2}+\frac{4M_{e}}{N}}}\right], \end{array} \end{array}$$

where SD = standard deviation. For very large data sets with *N* >> *M_e_*, SE simplifies to


(15) $$\begin{array}{c} SE({\hat{h}}^{2})\approx \frac{1.5c}{\sqrt{n}\;}. \end{array}$$

Note that *N* is the number of individuals in the reference population and *n* is the number of individuals in the validation population.

### Estimation procedure

1. Find an approximate number of independent chromosome segments (*M_e_*) using numbers provided by Pocrnic *et al*. (2016b) and formulas by Stam (1980) or by eigenvalue analysis as in Pocrnic *et al*. (2016a). For a large number of genotypes, calculate eigenvalues based on a sample, or use the Lanczos algorithm (Cullum and Willoughby 2002).
2. Select the validation generation. Small size would increase SE, whereas large size could average changing parameters.
3. Select the reference population prior to the validation generation. At best, the reference population needs to be larger than *M_e_*.
4. Calculate EBVs using phenotypes from the reference generation and genotypes and pedigrees from both reference and validation generations. Use known heritabilities and treat traits as uncorrelated.
5. Calculate predictivities within and across generations.
6. Recalculate heritabilities using equation (5).
7. Calculate accuracies using equation (2).
8. Calculate genetic correlations using equation (10).
9. Possibly use new heritabilities in Step 4 and repeat Steps 5–8. Iterate if changes are large.
10. If changes over time are of interest, repeat Steps 1–8 for multiple reference and validation generations.

### Testing

The formulas were tested in 2 ways. The first test evaluated the formulas in a large, simulated population of several genotyped generations under intensive selection. The simulation contained an artificially created fitness trait with a genetic correlation to a production trait declining each generation. The production trait was an individual trait, whereas the fitness trait was a composite trait with changes in the composite over time. The second test estimated heritabilities for large data sets based on predictivities or accuracies derived from predictivities and other quantities reported in published studies.

### Simulated data in multiple generations

Let $y_{i}^{j}$ be phenotypes of trait *i* (*i* = 1,2,3) for 40,000 individuals in generation *j* (*j* = 0, …, 6) with corresponding breeding values (*u*) and residuals (*e*). The initial generation was simulated using the following model:


$$\left[\begin{array}{c} y_{1}^{0}\\ y_{2}^{0} \end{array}\right]=\left[\begin{array}{c} u_{1}^{0}\\ u_{2}^{0} \end{array}\right]+\left[\begin{array}{c} e_{1}^{0}\\ e_{2}^{0} \end{array}\right]$$

with variances


$$\mathrm{Var}\left(\left[\begin{array}{c} u_{1}^{0}\\ u_{2}^{0} \end{array}\right]\right)=G⊗\left[\begin{array}{cc} 1 & 0\\ 0 & 1 \end{array}\right],\mathrm{Var}\left(\left[\begin{array}{c} e_{1}^{0}\\ e_{2}^{0} \end{array}\right]\right)=I⊗\left[\begin{array}{cc} 1.5 & 0\\ 0 & 9 \end{array}\right],$$

where **G** is a 40,000 × 40,000 genomic relationship matrix and **I** is an identity matrix. The variances were set so that the first trait mimics a production trait (*h*^2^ = 0.4) and the second trait mimics a fitness trait (*h*^2^ = 0.1). Data from generation *j* were analyzed by univariate genomic best linear unbiased prediction (GBLUP) using the parameters above and with the top 13 males and 10,000 females selected to be parents for generation *j* + 1, for an effective population size of 50. Trait 3 was created to have declining correlations with Trait 1 over generations:


$$u_{3}^{j}=\;\alpha_{j}[u_{2}^{j}-\beta u_{1}^{j}(\bar{u_{1}^{j}-u_{1}^{0}})],$$

where $\alpha_{j}$ is a scaling factor chosen so that $\mathrm{var}(u_{3}^{j})=1$ and *β* was chosen for correlations between Traits 1 and 3 to change by about −0.1 each generation.

The phenotype for Trait 3 was simulated as $y_{3}^{j}$:


$$y_{3}^{j}=\;u_{3}^{j}+e_{3}^{j},$$

where Var($e_{3}^{j}$) was the same as for Trait 2.

Simulations were performed by the AlphaSimR package version 3.4.4 (R Core Team) (Gaynor et al. 2021). The simulated genome contained 30 chromosomes with equal lengths of 100 cm each. Each chromosome harbored 100 quantitative trait nucleotides selected from the gamma distribution, and 50,000 biallelic SNP markers were used.

The following were calculated for Traits 1 and 3 and Generations 3–6:

1. Realized variances and realized genetic correlations for each generation.
2. Estimates of variance by a Bayesian approach via Gibbs sampling
3. Heritabilities and genetic correlations by predictivity using sliding windows of 3 generations, with the first 2 used as a reference population and the third as a validation population

The simulation was replicated 4 times. Estimation of breeding values and Gibbs sampling were done using the BLUPF90 suite of programs (Misztal *et al*. 2014).

### Test with real data sets from references

The formula for heritability was tested with data extracted from studies on milk yield of Holsteins (Cesarani *et al*. 2021), growth and fitness traits of pigs (Hollifield *et al*. 2021), and a growth trait of broiler chicken (Hidalgo *et al*. 2021). The information extracted from the studies included initial heritabilities used in the calculations, approximate number of genotyped animals with phenotypes in both reference and validation populations, calculated predictivity for the Holstein study, and calculated accuracies for the pig and chicken studies. Because the accuracies were computed based on unreported predictivities, those predictivities were recalculated as accuracy times the square root of heritability. Up to 3 generations of animals were included for pig and chicken data, and animals born after 2000 were included for Holsteins. The number of animals in the accuracy formula [equation (3)] was assumed to be the number of genotyped animals with phenotypes except for fitness traits of pigs, where fewer animals had phenotypes than genotypes; for those traits, the number of animals with phenotypes was used. The number of independent chromosome segments followed the empirical results of Pocrnic *et al*. (2016b), which was 15,000 for Holsteins and 5,000 for pigs and chickens.

## Results

### Simulated data with multiple generations

**Fig. 1.**
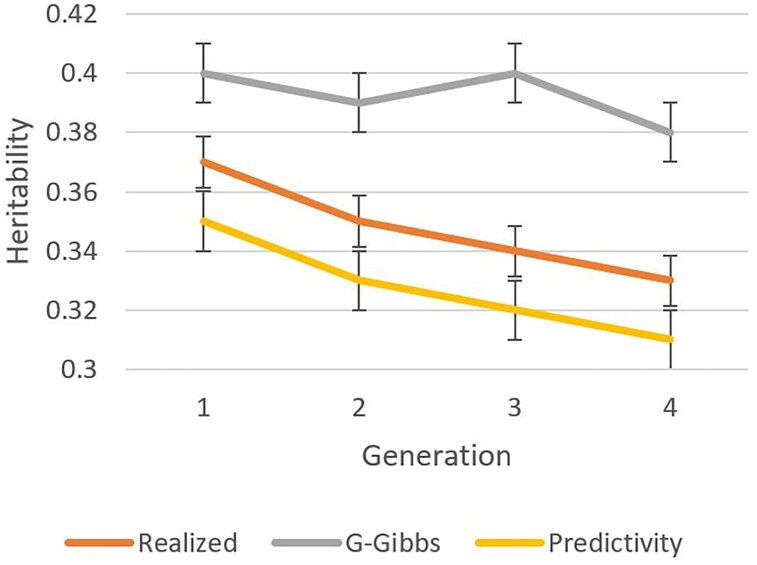
Heritability estimates for a simulated production trait by generation based on true breeding values (realized), genomic Gibbs sampling (G-Gibbs), and predictivity.

**Fig. 2.**
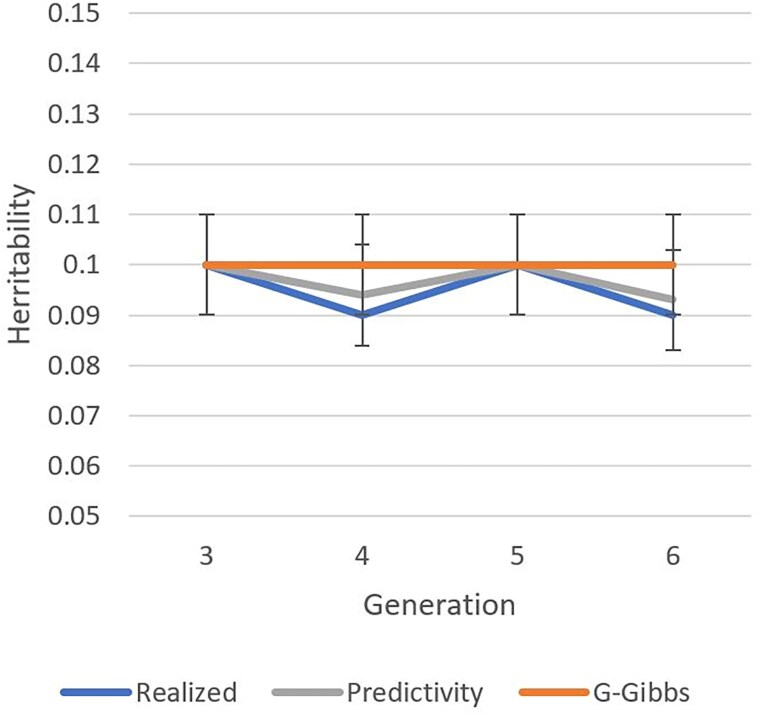
Heritability estimates for a simulated fitness trait by generation based on true breeding values (realized), genomic Gibbs sampling (G-Gibbs), and predictivity.

**Fig. 3.**
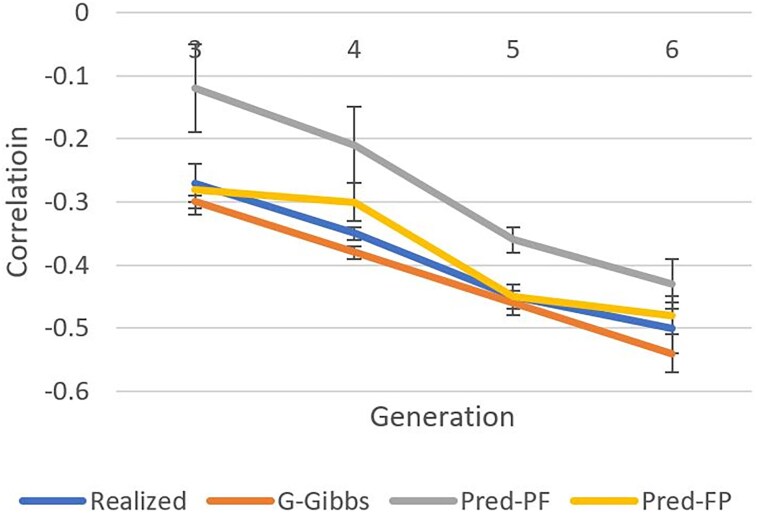
Genetic correlation estimates by generation based on true breeding values (realized), genomic Gibbs sampling (G-Gibbs), predictivity of production phenotype by fitness GEBV (Pred-PF), and predictivity of fitness genotype by production GEBV (Pred-FP).

Figures 1–3 show heritabilities and genetic correlations calculated from realized breeding values, by a Gibbs sampler, and by predictivity. The realized heritability for the production trait (Fig. 1) decreased with generation number, most likely because of the Bulmer effect.

Although it was 0.40 in Generation 0, it dropped to 0.37 in Generation 3 and to 0.33 in Generation 6. The estimates by Gibbs sampling were up to 0.06 higher. With estimation by REML, which has similar properties to the Bayesian method, Cesarani *et al*. (2021) found that estimates are biased under genomic selection, with biases smaller with more generations of data. Using more generations could have reduced the biases at the cost of averaging the heritability. The curve of heritability based on predictivity is almost parallel to the one based on realized breeding values, with a lag of about 0.02.

Heritabilities for the fitness trait (Fig. 2) were approximately constant because of the standardization of additive variance, and all heritabilities were similar regardless of the estimation method. One reason for the stability with all the methods is only indirect selection of the fitness trait by the production trait.

Realized genetic correlations (Fig. 3) declined by about 0.1 per generation as designed. Estimates based on Gibbs sampling were up to 0.04 lower. Estimates based on predictivity depended on the trait order of predictivity. Estimates were within 0.15 with predictivity of the production phenotype from fitness GEBV (called first) and within 0.05 with predictivity of the fitness phenotype from production GEBV (called second). In the first predictivity, prediction on fitness, a composite trait that changed over time, was based on 2 past generations, which created a lag of 1.5 generations; no lag was found in the second predictivity. When genetic correlations are computed by predictivity, a smaller bias can be expected by predicting the phenotype of a trait more likely to be changing (e.g. fitness) from a more stable trait (e.g. production). All SEs were low because of the large number of genotyped individuals.

Estimates by predictivity were derived assuming true initial heritabilities. To evaluate the effect of incorrect heritabilities on estimation, the simulation was repeated with heritabilities assumed to be 2 times higher and 2 times lower. In all cases, the effect on final estimates was minimal (≤0.01), which indicates that with large data sets the parameters estimated by predictivity are minimally affected by initial parameters.

Conducting Gibbs and possibly REML analyses on more than 1 generation could be informative. However, even with 1 generation, the 2-trait genomic relationship matrix (80,000 × 80,000) was too large for REML in the BLUPF90 suite to handle. Computation was possible using a program for a Bayesian approach by Gibbs sampling in the same suite because that program was optimized by storing single-trait versions of coefficients and relationship matrices and reconstructing multiple-trait equations on the fly. In this study, parameter computations for predictivity took about 30 minutes and for Gibbs sampling about 40 hours. With more generations or genotypes, computing would increase cubically with REML and quadratically with the Bayesian approach. On the other hand, estimation using predictivities with GEBVs computed by iteration on data incurs only a linear cost.

### Heritability calculations

Table 1 shows details of heritability calculations for Holsteins and 2 traits of pigs and chickens. Holsteins had the largest number of animals with phenotypes and genotypes (580,000) and the largest number of validation animals (381,000).

**Table 1.** Description of real data sets for estimating heritabilities.

	Species/trait			
	Holsteins/milk	Pigs/fitness	Pigs/growth	Chicken/growth
Number of animals with phenotypes and genotypes in reference population (*N*)	580,000	12,000 (Generations 5–7)	20,000 (Generations 5–7)	∼100,000
Number of animals in validation population (*n*)	381,000	2,000 (Generation 8)	11,000 (Generation 8)	∼40,000
Initial heritability	0.35	0.05	0.21	0.30
Assumed *M_e_*	15,000	5,000	5,000	5,000
Calculated accuracy		0.40	0.80	0.58
Predictivity	0.55	0.09	0.36	0.31
Calculated heritability	0.33	0.06	0.26	0.14

Heritability calculated from the formula was 0.33, close to the initial heritability of 0.35 from the study of Cesarani *et al*. (2021). The next largest data set was for a growth trait of chickens, which included about 100,000 animals with genotypes and phenotypes. Although Hidalgo *et al*. (2021) assumed a heritability of 0.30 based on earlier studies, the calculated heritability was lower (0.14). Based on more recent studies of the data providers (Vivian Breen, personal communication), a heritability of 0.13 is currently used. A higher heritability growth trait and a lower heritability fitness trait were examined for pigs. The initial heritability was 0.21 for the growth trait and 0.05 for the fitness trait (Hollifield *et al*. 2021), and the calculated heritability was slightly higher (0.26) for the growth trait and almost the same (0.06) for the fitness trait.

## Discussion

The proposed methods allow an inexpensive estimation of heritability and genetic correlation trends by time intervals. For each interval, predictivity is calculated within and across traits. After assuming the number of independent chromosome segments, heritabilities, genetic correlations, and their SEs are calculated. The trends of genetic parameters would allow extrapolating changes and modifying the selection index to avoid future problems. The advantage of the proposed methods is low computing cost for any model with any number of genotyped individuals, because the only computationally intensive step is the genetic evaluation.

Questions can be raised about the robustness of the formulas, however. The formula of Daetwyler *et al*. (2008) was derived assuming only animals with genotypes and phenotypes and under ideal conditions: no genotyping errors and, indirectly, an unlimited number of markers. Therefore, in practical cases, it may give an inflated accuracy estimate, e.g. as found by Erbe *et al*. (2013). Field data usually have additional information from nongenotyped animals, which is considered in single-step GBLUP (Aguilar *et al*. 2010). Nevertheless, the genotyping strategy may be suboptimal, and both genotyping and pedigree errors exist. Subsequently, the accuracy would be smaller than in idealized conditions. When the number of genotyped animals is much larger than *M_e_*, a compromise choice for *N* would be the number of animals with both genotypes and phenotypes.

Another value required by the formula of Daetwyler *et al*. (2008) is *M_e_*. Such a number can be derived in several ways: for example, 4*N_e_L*, where *N_e_* is the effective population size and *L* is the length of genome (Stam 1980), or the number of eigenvalues that explain 98% of the variation in the genomic relationship matrix (Pocrnic *et al*. 2016a). Both studies suggest an *M_e_* of 5,000–15,000 for farm animals, and unpublished results indicate <2,000 for crosses of inbred lines in plants. With a large number of genotyped individuals, the change in accuracy in the formula of Daetwyler *et al*. (2008) with increased *N* or *M_e_* is small. Assuming that having many individuals corresponds to ≥4*M_e_*, the formula for heritability can be applied with reference populations of at least 20,000–60,000 genotyped individuals for animals, about 8,000 for plants, and 1.5 million for humans (*N_e_* ≥ 3,000).

A serious concern is the susceptibility of the predictivity to selection bias (Bijma 2012). A substantial bias was observed in earlier studies with limited genotyping (Lourenco *et al*. 2015). In a recent study (Abdollahi-Arpanahi *et al*. 2022), accuracies by predictivity and the linear regression (LR) method (Legarra and Reverter 2018), which is considered to be resistant to selection bias, were nearly identical, indicating that selection biases with the predictivity formula are low with widespread genotyping. The formulas for the genetic correlations could possibly be incorporated into the LR method to take advantage of simpler computing and better statistics. For example, the adjustment for fixed effects must be explicitly made in the predictivity formula, but such an adjustment is automatic in the LR method.

Estimation by predictivity is affected by the applicability of the formula by Daetwyler *et al*. (2008). The sensitivity of that formula to the value of *M_e_* and discrepancies with single-step GBLUP is smaller when accuracy from that formula is high (e.g. >0.7). In such a case,


$$N:\;\sqrt{\frac{Nh^{2}}{Nh^{2}+M_{e}}}>0.5.$$

Then approximately, $Nh^{2}>M_{e}/3$, and $N>M_{e}/3h^{2}$. Assuming *M_e_* ≈ 15,000 (cattle) and a medium heritability trait with *h*^2^ ≈ 0.3, the minimum size of the reference population would be 16,000. The size would increase with lower heritability traits and would be 100,000 for a trait with *h*^2^ ≈ 0.05. The size would be about 3 times smaller for pigs and chickens with *M_e_* ≈ 5,000.

Estimation by predictivity can be done whenever the predictivity can be calculated. The formula for predictivity was derived from a very simple model [equation (1)] that included only 1 random effect. In practice, more complicated models are used by adjusting the phenotype in equation (1) for all effects except the random effect of interest. Such an adjustment will not work well in models with correlated random effects (e.g. in a random-regression model).

In the simulation, the estimates of the same correlations depended on the order of traits in the predictivity formula. A general recommendation would be to use predictivity with phenotypes of a fast-changing (or composite) trait and EBV of a slow-changing trait. In practice, the difference between the two correlations well more than standard errors could help pinpoint faster-changing traits.

In the simulation, the process of creating the changing trait was artificial, and the model of analysis did not account for the changes. It would be desirable to create a realistic model, e.g. based on a resource allocation theory that would allow for changes over time (Van der Waaij 2004; Bouquet *et al*. 2022). Possibly such a model could be a generalization of a recursive model (Gianola and Sorensen 2004), which allows for an unsymmetrical relationship among traits.

For complicated models, using established methods such as REML or Bayesian via Gibbs sampling (Sorensen and Gianola 2002) with random regression on the year of birth (Tsuruta *et al*. 2004) may be the only choice. Although those methods are prohibitively expensive with large models, computing refinements may make them applicable for larger although not necessarily complete data sets. For example, Matilainen et al. (2019) presented a Monte Carlo version of REML, which does not require storage or inversion of mixed-model equations. The method was applied to multiple-trait analyses of 3 million animals, although computation took a long time (39 days) and the method was not yet applicable to genomic data (Bonifazi *et al*. 2020).

## Conclusions

Genetic heritabilities and correlations can be estimated in large genomic models for any slice of data by using predictability within and across traits. The computing requirements are similar to running genetic evaluations. The proposed method can be applied for estimation of genetic parameters with large data sets where other methods are not computationally feasible. In particular, it can be used as a tool in commercial genetic evaluations to monitor changes in parameters over time, e.g. every cycle of selection or yearly. If fast changes are detected, particularly indicating increased antagonism among important traits, the updated parameters can be applied in the genetic evaluation and in calculating weights in the selection index. In addition, the method can help identify “composite” traits that change faster over time by examining differences in estimates of genetic correlations based on the two estimates of genetic correlations. Such information may be useful to truncate old data that is no longer useful for genetic prediction of the newest generation. The proposed methods are mainly applicable to large data sets and extensive genotyping where predictivity is not affected by selective genotyping.

## Supplementary Material

iyaf066_Supplementary_Data

## Data Availability

Programs in R to simulate the data in this study are available as Supplementary Files 1 and 2 at GENETICS online. Supplemental material available at GENETICS online.
